# Implementation of a voluntary deep inspiration breath hold technique (vDIBH) using BrainLab ExacTrac infrared optical tracking system

**DOI:** 10.1371/journal.pone.0195506

**Published:** 2018-05-10

**Authors:** Edy Ippolito, Michele Fiore, Alessia Di Donato, Sonia Silipigni, Carla Rinaldi, Patrizia Cornacchione, Erminia Infusino, Cristina Di Venanzio, Carlo Greco, Lucio Trodella, Sara Ramella, Rolando Maria D’Angelillo

**Affiliations:** Radiation Oncology, Università Campus Bio-Medico, Rome, Italy; National Institute of Health, ITALY

## Abstract

**Background:**

Voluntary deep inspiration breath hold technique (vDIBH) is considered as the key to achieving the widest cardiac sparing in whole breast irradiation. Several techniques have been implemented to achieve a reproducible, fast and friendly treatment. The aim of the present study is to implement vDIBH using the ExacTrac (BrainLAB AG, Germany) monitoring system.

**Methods:**

Women with left-sided breast cancer, younger than 50 years or with cardiac disease, underwent whole breast RT with vDIBH using the ExacTrac (BrainLAB AG, Germany) monitoring system. Simulations were performed with patients positioned supine on a breast board with both arms raised above the head. Five optical markers were placed on the skin around the border of the left breast gland and their position was referenced with ink marking. Each patient received a training session to find the individual deep inspiration level. Finally, a vDIBH CT was taken. All patients were also studied in free breathing (FB) in order to compare the dose distribution for PTV, heart and left anterior descending coronary artery (LAD). Pre-treatment verification was carried out through the ExacTrac (BrainLAB AG, Germany) system and verified with electronic portal imaging (EPI). Moreover, daily real time EPIs in during modality (captured during the beam delivery) were taken in order to check the reproducibility.

**Results:**

34 patients have been evaluated and 30 were eligible for vDIBH. Most patients showed small setup errors during the treatment course of below 5 mm in 94.9% of the recorded fields. Mean Displacement was less in cranio-caudal direction. Mean intra-fraction displacement was below 3 mm in all directions. vDIBH plans provided better cardiac dosimetry.

**Conclusions:**

vDIBH technique using ExacTrac (BrainLAB AG, Germany) monitoring system was applied with good reproducibility.

## Introduction

Adjuvant radiotherapy after breast conserving surgery reduces the risk of loco-regional recurrence and is at present time widely used as standard of care [1]. The increasing number of screened patients with early disease, along with improvements in oncological treatment, has led to a greater number of women, at risk of developing chronic toxicities, surviving for many years after diagnosis[2].

In particular, there is data regarding patients with left-side breast cancer, where radiotherapy can increase the risk of late heart disease. Radiation induced heart toxicity could involve different cardiac structures such as vessels, myocardium, pericardium and valves, but coronary artery disease (CAD) or perfusion defect cardiomyopathy are the more common [3].

Darby et al, [4] have shown that the risk of heart disease increases linearly with increased cardiac exposure (7.4% per Gy of mean heart dose). The authors also reported that if a patient, without cardiac comorbidities, receives a dose of 2 Gy to the heart from left breast radiotherapy, the absolute 30 year risk of death from radiation related ischemic heart disease would be less than 0,1% [5]. Moreover, a meta-analyses [6] recorded a link between cardiac deaths following breast radiotherapy and the volume of the heart receiving 5 Gy.

A recent population-based study by Boero et al, [7] evaluated the effect of current radiation practice on ischemia-related events and procedures on a total of 29102 patients diagnosed from 2000 to 2009. Female patients with a history of cardiac disease who underwent radiation therapy for left-sided breast cancer had an increased risk of percutaneous coronary intervention (PCI) after radiation therapy with a 10-year cumulative incidence of 5.5% (95% confidence interval [CI] 4.9%-6.2%) and a significantly increased risk of cardiac mortality if treated with PCI.

Consequently, there is a need in clinical practice for new strategies that allow for a reduction in cardiac exposure from left-sided breast cancer radiotherapy. Several methods to reduce radiation doses to the heart have been evaluated, including: 1) novel and more sophisticated technologies in the delivery (i.e., IMRT, VMAT, arc therapy, proton beam radiotherapy); 2) partial breast irradiation (reducing the conventional target of the whole breast); and 3) respiratory gated radiotherapy.

Gating techniques are often preferred, mainly in deep inspiration, because when a patient inhales, the heart turns away from the chest wall moving posteriorly, medially and inferiorly and this increases the distance between the heart and the chest wall [8].

A large decrease in heart V50%, up to 80–90% and in median dose delivered to the left anterior descending coronary artery have been demonstrated through deep inspiration breath hold [9]. Furthermore a reduction in heart V20Gy and V40Gy and mean heart dose was also reported in a similar study [10].

There are several techniques available to monitor breath hold during treatment delivery. The active breathing control (ABC) technique uses a computer-controlled valve that ensures a uniform inhaled volume. A similar technique uses respiratory gating with chest wall sensors to drive delivery of radiotherapy based on thorax expansion [11–12].

A recent randomized trial comparing the ABC technique with voluntary breath hold found no differences in terms of reproducibility and heart or descending artery doses [13].

To date, there is no sufficient data to recommend one breath hold technique over another, although it is well known that deep inspiration breath hold is able to achieve the greatest cardiac sparing.

Based on the above considerations, we clinically implemented a voluntary deep inspiration breath hold (vDIBH) radiation program for patients with left-sided breast cancer using an infrared optical tracking system, ExacTrac (BrainLAB AG, Germany), evaluating tissue sparing and reproducibility of technique.

## Materials and methods

Patients with left-sided breast cancer undergoing whole breast irradiation with vDIBH treatment were evaluated for set-up reproducibility and breath hold monitoring using ExacTrac (BrainLAB AG, Germany). The study was developed according to the principles outlined in the Declaration of Helsinki. This study was reviewed and approved by the Campus Biomedico University institutional review board. All patients agreed to the treatment and gave their written informed consent prior to radiation therapy.

### Radiotherapy technique

Simulations have been performed with patients positioned supine on a breast board with both arms raised above the head. A free breathing (FB) CT scan was taken. Thereafter, in the same position, each patient received a training session to establish the individual deep inspiration level. Patients who were not able to maintain a stable deep inspiration breath hold for at least 20 seconds were excluded. Five optical markers were placed on the skin around the border of the left breast gland and their position was referenced with ink marking and a vDIBH CT scan was taken. The scan was extended from the jugular notch to 5 cm below the lower edge of the breast with a scan interval of 5 mm. The target volume, the heart, left anterior descending artery (LAD), contralateral breast and ipsilateral lung were manually contoured on each CT slice by a radiation oncologist with at least 5 years experience following the RTOG guidelines [14] and heart atlas published by Feng et al [15]. Subsequently, all contours were independently reviewed by another radiation oncologist with more than 10-years experience in breast cancer. The clinical target volume (CTV) was defined as the entire breast, excluding the outer 4 mm from the skin surface. The planning target volume (PTV) was defined as CTV + 5 mm in the direction of the chest wall. The total prescribed dose was 50 Gy at the isocentre in accordance with the International Commission on Radiation Units Measurement.

Treatment plans consisted of a simple wedged tangential plan (with gantry angles optimized to match divergence of the posterior edges of the beam) to avoid contralateral breast irradiation and to minimize the ipsilateral lung and heart area. Moreover, in some cases a field in field technique was also applied. The Eclipse (Varian Medical Systems, Palo Alto, CA) Triple A dose calculation was used for planning.

According to the Danish Breast Cancer Cooperative Group [16], the following organ at risk (OAR) constraints were applied: LAD Dmax = 20Gy; mean heart dose (Dmean) ≤3 Gy, percentage of the ipsilateral lung volume receiving at least 20 Gy (V20Gy< 20 Gy). All evaluated plans either in free breathing or in vDIBH reached these constraints.

### ExacTrac (BrainLAB AG, Germany) infrared optical tracking

**Fig 1:**
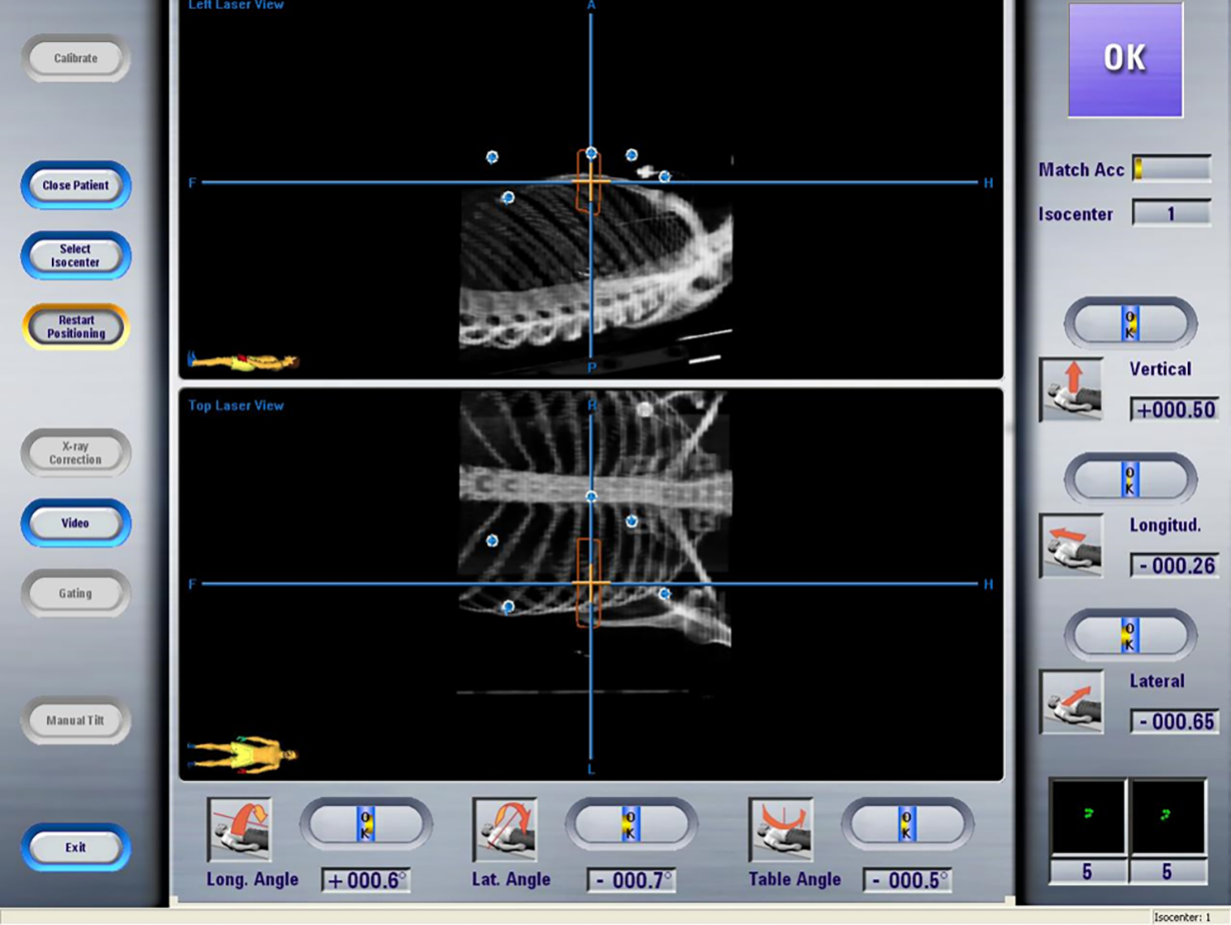
BrainLab ExacTrac monitoring system for patients set-up.

ExacTrac (BrainLAB AG, Germany) uses two infrared cameras mounted in the ceiling of the linac vault to track optical markers placed on skin landmarks. A calibration procedure was used in order to determine the position of any passive optical marker relative to the isocentre. The system was calibrated to a common isocentric reference frame, providing sub-millimetric accuracy in 3D markers localization. This system was the reference system used to set up patients during the vDIBH in both simulation and treatment rooms (Fig 1) as the infrared camera receives and reflects infrared rays emitted from surface markers placed around the border of the left breast gland. No visual feedback was provided.

### Set-up verification and breath-hold monitoring

**Fig 2:**
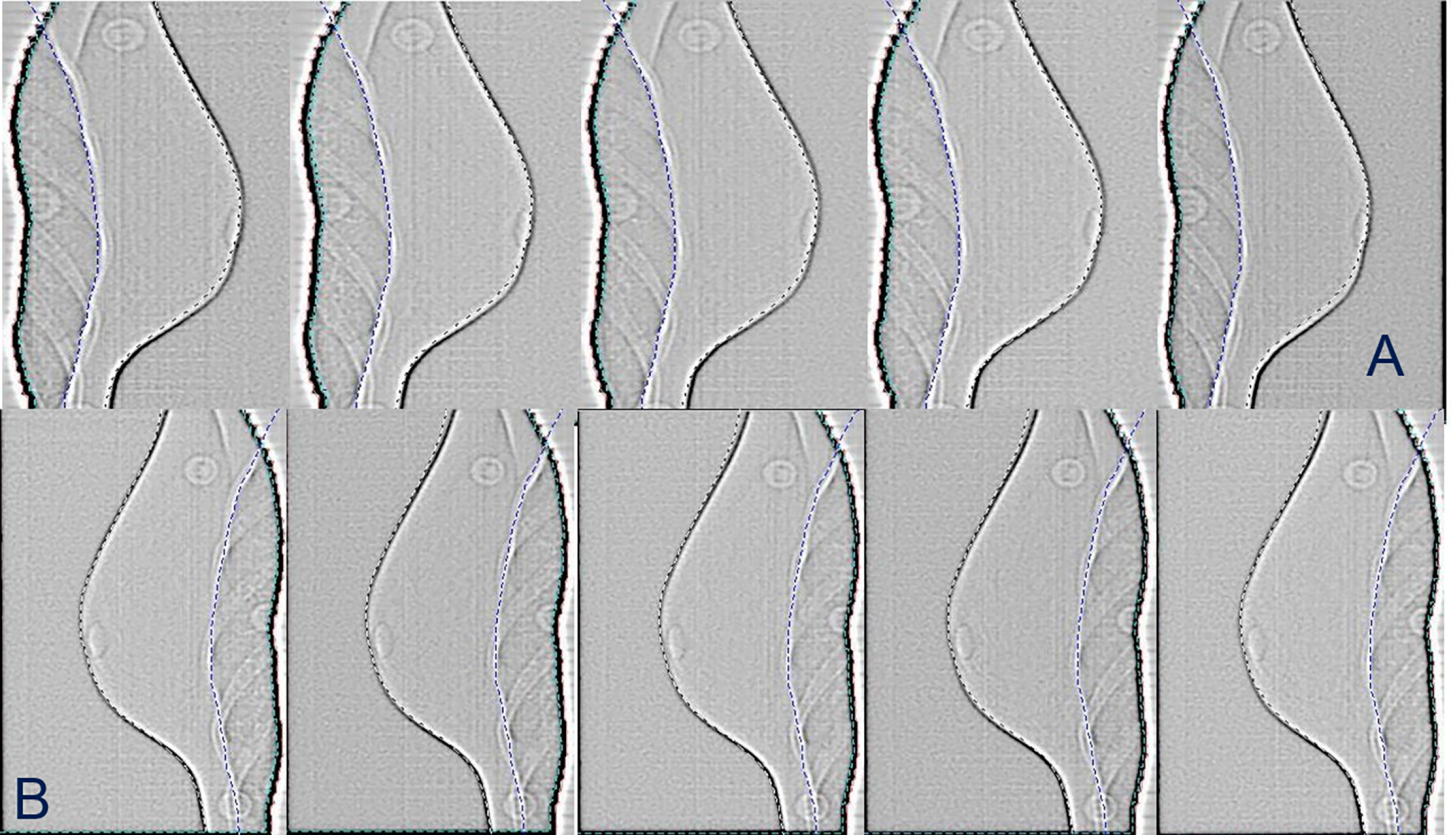
**Continuous portal imaging for lateral (A) and medial (B) fields**.

The ExacTrac Infrared monitoring device tracked the position of the patient as well as breath hold; particularly, this system was able to reveal if breath hold level was different from the one achieved during simulation. Before each treatment, tangential fields (lateral and medial fields) were taken with electronic portal imaging (EPI). In order to evaluate intra-fraction motion, continuous portal imaging (captured during beam delivery) of both lateral and medial fields were also obtained (Fig 2). These images were compared to the Digitally Reconstructed Radiographs (DRRs) using the image review application (ARIA v.13, Varian Medical Systems).

### Statistical analysis

Comparison between the dosimetric data of the two techniques (FB and vDIBH) was performed by means of paired t-test.

A four-parameter model was used to analyze the setup error of a population of patients, with each patient having her individual setup error. The four-parameter model separates the total distribution of positioning corrections (which consists of all fractions for all patients) into a distribution of patient-specific systematic setup errors and a distribution of patient-specific random setup errors. Systematic error can be defined as the mean (Σ) of positioning corrections for that patient, and random error can be defined as the standard deviation (σ). The formalism described by Yan et al. [17] was used to estimate the inter-fraction set-up error quantified as mean displacement (MD) systematic (Σ) and random (σ) errors of the mean chest wall position of lateral and medial field before treatment. This represents the set-up treatment accuracy. The intra-fraction motion was represented by mean displacement (MD) and Σ and σ errors of the mean chest wall position during delivery of lateral and medial field. An inter-field shift was also calculated as the difference in mean error of the chest wall between field 1 and 2 during beam delivery. This latter data represents the accuracy in reproducibility of the internal gating. All the errors were evaluated in three directions (medio-lateral, M-L; cranio-caudal, C-C, antero-posterior, A-P).

The statistical analysis was performed using SYSTAT, version 12.0 (SPSS, Chicago, IL).

## Results

Thirty-four consecutive patients affected by left sided breast cancer were recruited for this study. Four patients were not able to maintain a stable breath hold for at least 20 seconds and were excluded from the study. Median age was 53 years (range 31–78). Patients’ characteristics are listed in Table 1.

**Table 1:** Patients’ characteristics.

	N
Patients	30
Age, years median	53
Age, years range	31–78
Cardiovascular comorbidities	
Yes	3 (10%)
Not	27 (90%)
Previous antracyclines chemotherapy	
Yes	11 (36.7%)
Not	19 (63.3%)
Previous trastuzumab immunotherapy	
Yes	7 (23.3%)
Not	23 (76.7%)

### Dosimetric comparison between FB and vDIBH treatment plans

Quantitative dosimetric comparisons between FB and vDIBH plans for V95%, ipsilateral lung V20Gy, LAD artery, heart and ipsilateral lung are shown in Table 2. Target coverage (V95%) was improved in vDIBH plans (97.8%) vs FB (96.5%; p<0.001). LAD Dmax and mean heart dose were improved by means of vDIBH over FB with a dose reduction of 37% and 24%, respectively. (p<0.001)

**Table 2:** Dosimetric data according to FB and vDIBH (target coverage and cardiac dosimetry).

	FB treatment plans	vDIBH treatment plans	p value
PTV V95% (%)			
Mean	96.53	97.81	<0.001
SD	1.98	1.98	
LAD (Dmax, Gy)			
Mean	18.02	11.41	<0.001
SD	5.12	6.13	
Heart (Dmean, Gy)			
Mean	2.01	1.52	0.001
SD	1.01	0.49	

FB = free-breathing; vDIBH = voluntary deep inspiration breath hold; PTV V95% = volume of PTV receiving 95% of prescribed dose; LAD = left anterior descending artery

### Set-up data and breath-hold monitoring

Most patients showed small setup errors during the treatment course of below 5 mm in 94.9% of the recorded fields. Mean Displacement was less in C-C direction. The intra-fraction motion was limited with a mean displacement being below 3 mm in all directions.

The inter-field shift (mm) between field 1 and 2 during beam delivery was 0.67 mm in M-L, 0.24 mm in C-C and 0.29 mm in A-P direction. Table 3 shows population MD, Σ and σ in M-L, C-C, V-L directions, estimated using EPI for set-up errors and intra-fraction motion. The mean time needed to treat patient for each single fraction was 6.86 (SD = 3.11) minutes.

**Table 3:** Population mean displacement (MD), systematic (Σ), and random (σ) in 3-dimensions for interfraction set-up errors and intrafraction motion.

		Interfraction set-up errors (mm)		Intrafraction motion (mm)	
		Field 1*	Field 2°	Field 1*	Field 2°
Medio-lateral (M-L)	MD	2.1	2.0	2.2	2.3
	Σ	1.3	1.9	1.3	1.2
	σ	2.0	1.7	0.9	1.2
Cranio-caudal (C-C)	MD	1.6	1.9	2.7	2.9
	Σ	2.0	1.6	1.3	1.6
	σ	0.8	1.1	1.3	1.5
Antero-posterior (A-P)	MD	2.7	2.4	2.5	2.4
	Σ	0.8	1.1	1.4	1.3
	σ	1.7	2.0	1.0	1.1

*Field 1: Latero-medial field

°Field 2: Medio-Lateral field; all treatment was delivered with a classical tangential technique

## Discussion

Breath hold technique is a widely recognized heart sparing technique. Voluntary breath hold techniques (vDIBH) could be offered to a large portion of left-sided breast cancer patients to reduce late cardiac sequelae. In this study we clinically implemented a vDIBH radiation program for patients with left-sided breast cancer using an infrared optical tracking system, ExacTrac (BrainLAB AG, Germany), evaluating the reproducibility of technique.

These dosimetric advantages of vDIBH have been confirmed in the UK HeartSpare study where a very simple technique, operator’s visual feedback only, with no additional equipment to active breathing coordinator technique was applied [13].

More sophisticated techniques have been developed such as ABC or RPM system. The last technique showed a 80–90% reduction of the heart V50 for left-sided cancers which authors stated could translate into a reduction of cardiac mortality probability from 4.8% for FB techniques to 0.1% for DIBH [8–10]. A reduction in maximum LAD dose was also documented [18]. Recently optical surface scanning of the patients was also used for DIBH. Schönecker et al reported that the maximum doses to the heart and LAD were reduced by 59% and 75%, respectively in 13 patients using a laser surface scanner and an optical surface scanner systems for DIBH [19].

Similarly, in our study, a significant reduction in both cardiac constraints were recorded. In particular, Dmax to LAD artery received a huge benefit with a reduction from 18 Gy of FB to 11.4 Gy of vDIBH.

As far as we know, this is the first study using an infrared optical tracking system, ExacTrac (BrainLAB AG, Germany) for the treatment of left sided breast cancer with vDIBH technique.

Data on reproducibility seems promising as most patients showed small setup errors during the treatment course (below 5 mm in 94.9% of the recorded fields) with the intra-fraction motion being below 3 mm in all directions (mean displacement). Larger displacements were observed in anterior-posterior direction for set-up errors (2.7 and 2.4 mm for both fields) and in cranio-caudal direction for intra-fraction motion (2.7 and 2.9 mm for both fields).

For set-up errors this may be due to difficulties in achieving the correct breath hold level during set-up due to the lack of a visual feedback; alternatively, for intra-fraction residual motion, this may be due to the position of the optical markers, even if the larger margins in this direction, typical of tangential field, could reduce the clinical impact of such errors.

Lutz et al. [20] investigated the position and residual motion of the chest wall of 58 breast cancer patients during RPM based vDIBH. In particular, the study compared the DIBH reproducibility in a group of patients in which the external marker block was placed 5 cm to the right at the level of the xiphoid process (group 1) or placed medially on the inferior part of the sternum (group 2). A tendency for a smaller set-up error was observed for group 2. Similarly, Skytta T et al. [21] demonstrated that reproducibility of the BHL can be improved by placing the marker block on the sternum. This data partially supports the position of the optical markers used in our study. In fact, we believe we can improve reproducibility by modifying the position of the markers at the upper and lower border by placing them more medially along the sternum in order to better represent chest wall stability.

In the UK HeartSpare study [13] reproducibility data of vDIBH derived from EPI verification was similar to ours. In this study, radiographers were instructed to check the light fields on the ink marked breath hold field by zoomed room cameras. This method compared with the ABC breath hold reduced costs and was less time consuming. In the same way, the adoption of ExacTrac (BrainLAB AG, Germany) for vDIBH was easily conducted by patients (30/34 patients, 88%, were able to perform the treatment) and it provided visual feedback to operators with a quantitative measurement better than the qualitative one’s observed in the UK HeartSpare study.

## Conclusions

In conclusion, delivering vDIBH with the Brainlab ExacTrac monitor system is feasible and does not seem inferior to other techniques employing vDIBH in terms of reproducibility.
